# Exon 11 homozygous mutations and intron 10/exon 11 junction deletions in the* KIT* gene are associated with poor prognosis of patients with gastrointestinal stromal tumors

**DOI:** 10.1002/cam4.3212

**Published:** 2020-07-22

**Authors:** Yan‐Ying Shen, Xin‐Li Ma, Ming Wang, Chun Zhuang, Bo Ni, Lin Tu, Qiang Liu, Wen‐Yi Zhao, Hui Cao

**Affiliations:** ^1^ Department of Gastrointestinal Surgery Renji Hospital School of Medicine Shanghai Jiaotong University Shanghai P.R. China; ^2^ Department of Pathology Renji Hospital School of Medicine Shanghai Jiaotong University Shanghai P.R. China

**Keywords:** gastrointestinal stromal tumor, homozygous mutation, KIT, prognosis

## Abstract

**Background:**

Gastrointestinal stromal tumors (GISTs) with different types of mutations exhibit different clinical characteristics and prognosis. This study aimed to evaluate the prognostic value of mutations in *KIT* and *PDGFRA* in a large‐scale cohort of GIST patients with current therapy including surgery and imatinib.

**Methods:**

A total of 1163 patients diagnosed with GISTs between January 2006 and December 2018 were enrolled in this study. Mutation analysis was performed for exons 9, 11, 13, and 17 of *KIT* and exons 12 and 18 of *PDGFRA*. Mutations were grouped into 12 categories according to the gene, exon, and involved codons; they were analyzed considering the clinical characteristics, disease‐free survival (DFS), and overall survival (OS) of patients with GISTs.

**Results:**

In low‐risk GISTs, we identified two predictors of worse DFS: tumor origin in the rectum and *KIT* exon 11 deletion involving two or more codons. In high‐risk GISTs treated with *R*_0_ resection and imatinib, patients with *KIT* exon 11 homozygous mutations and *KIT* intron 10/exon 11 junction deletions demonstrated the highest recurrence rate, indicating that these mutations can be independent prognostic factors of DFS. The presence of *KIT* exon 11 homozygous mutations also independently influenced OS.

**Conclusion:**

Low‐incidence mutations such as *KIT* exon 11 homozygous mutations or intron 10/exon 11 junction deletions in GISTs should be carefully evaluated to explore novel treatment strategies, as tumors with these mutations have a high recurrence rate and a very poor prognosis after surgery followed by imatinib adjuvant treatment.

## Graphical abstract

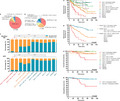

## 1 INTRODUCTION

Gastrointestinal stromal tumors (GISTs) are the most common mesenchymal tumors of the digestive tract, accounting for 1%‐4% of all malignant gastric tumors.^1^ GISTs are clinically heterogeneous, ranging from clinically benign, small to medium‐sized tumors to frank sarcomas. The following four clinicopathological features form the basis of the currently used risk‐stratification systems: tumor size, mitotic activity, primary tumor location, and the presence of tumor rupture.^2^, ^3^ The AFIP (American Forces Institute of Pathology) scheme proposed by Miettinen in 2006 and the modified NIH (National Institutes of Health) scheme proposed by Joensuu in 2008 are the two most commonly used risk assessment systems.^2^, ^3^

GISTs are characterized by gain‐of‐function mutations in the *KIT* or *PDGFRA* genes.^1^, ^4^ Many types of *KIT* and *PDGFRA* mutations have been observed in GISTs. GISTs with different types of mutations exhibit different clinical characteristics, therapeutic effects, and prognosis. *PDGFRA* mutant GISTs show a comparatively benign clinical behavior,^5^ while GISTs with *KIT* exon 9 duplications and exon 11 deletions, in particular deletions involving codons 557 or 558, are associated with higher malignant potential and shorter disease‐free survival (DFS) after surgery.^6^, ^7^ In addition, exon 11 mutant GISTs are usually sensitive to imatinib. In fact, imatinib therapy for 3 years after surgery significantly improved the prognosis of patients with GISTs with *KIT* exon 11 deletion.^8^, ^9^

However, in clinical practice, some GISTs carry the *KIT* exon 11 mutation but still show tumor metastasis and rapid growth after surgical resection and imatinib therapy. Thus, GISTs with *KIT* exon 11 mutations are heterogeneous tumors and further subdivision is necessary.

Recently, in our center, we found two groups of GISTs that showed aggressive biological behavior: one group was GIST with a *KIT* homozygous mutation and the other group was GIST with a *KIT* deletion encompassing the noncoding region in intron 10 and the coding region in exon 11. Both types of GISTs usually have a high mitotic index, are usually rated as high risk, and tend to metastasize at the time of detection. To the best of our knowledge, these particular mutations had only been detected in a few previous studies.^10^, ^11^, ^12^, ^13^, ^14^ However, the prognosis of patients with GISTs with *KIT* homozygous mutation and intron 10/exon 11 junction deletions has still not been reported. Moreover, it is unclear whether the prognosis of GIST patients with these particular mutations is worse than that of GIST patients with other mutation types such as exon 9 mutation or exon 11 deletion involving 557 and 558.

Therefore, in this study, we made a detailed distinction between the genotypes of GISTs and divided them into 12 categories. This study aimed to evaluate the prognostic value of *KIT* and *PDGFRA* mutation subtypes in a large‐scale cohort of Chinese patients with GIST after treatment with the current therapy including surgery and imatinib treatment.

## 2 METHODS

### 2.1 Clinical data of patients

Patients diagnosed with GIST between January 2006 and December 2018 at Renji Hospital, School of Medicine, Shanghai Jiaotong University, Shanghai, China, with available clinical data and mutational status were included in the study. This study was approved by the ethics committee of Renji Hospital, School of Medicine, Shanghai Jiao Tong University, Shanghai, China. Clinicopathological data were obtained by performing a retrospective review of all available medical and histopathological records. Preoperative imaging data and surgical records were used to determine whether the patient had localized or metastatic GIST. Patients with multiple or recurrent GISTs or other malignant tumors were excluded. Follow‐up information was obtained during regular outpatient visits or via phone calls to the patients. Disease‐free survival (DFS) was the time interval between the date of surgery and the date of relapse. Overall survival (OS) was the time interval between the date of surgery and death resulting from any cause.

### 2.2 Histopathological evaluation

The histopathological diagnosis of GIST was confirmed by two pathologists before the patients were included in the study. Diagnostic criteria were based on the Chinese consensus guidelines for diagnosis and management of GIST in 2017.^15^ For resected localized GISTs, the largest tumor diameter and mitotic count per 50 high‐power fields (HPFs) were evaluated as recommended by the international criteria.^2^, ^3^ Risk stratification was performed according to the modified NIH scheme and the AFIP scheme 2, 3. *R*_0_ resection is defined as no tumor residue under the microscope, *R*_1_ resection as tumor residue under the microscope, and *R*_2_ resection as tumor residue visible to the naked eye.

### 2.3 Mutation analysis

Mutation analysis was performed with genomic DNA isolated from formalin‐fixed paraffin‐embedded tissues by macrodissection and standard procedures.^14^ Macrodissection was made to ensure that tissue samples used for genetic testing contained more than 90% of the tumor. *KIT* exons 9, 11, 13, and 17 and *PDGFRA* exons 12 and 18 were amplified. PCR reaction conditions and primer sequence are shown in Table S1. The amplification products were purified, followed by bidirectional Sanger sequencing by an ABI 3130 Sequencer (Applied Biosystems, Inc). The mutation was considered homozygous when only the mutant allele was identified or when the height of the normal peak was only 30% or less compared to that of the mutant peak in the sequence diagram.

### 2.4 Statistical analysis

Frequency tables were analyzed using the *χ* ^2^ test. Survival analysis was conducted using the Kaplan–Meier method, and survival between groups were compared by the log‐rank test. Univariable and multivariable Cox regression models were used to determine associations between variables of interest (age, sex, tumor site, tumor size, mitosis, and tumor genotype) and outcome variables (DFS and OS). All reported *P* values were two‐tailed and statistical significance was defined as *P* < .05. Analyses were performed using Statistical Package for the Social Sciences (SPSS) version 16.0 (SPSS, Inc).

## 3 RESULTS

### 3.1 Clinicopathological characteristics

**FIGURE 1:**
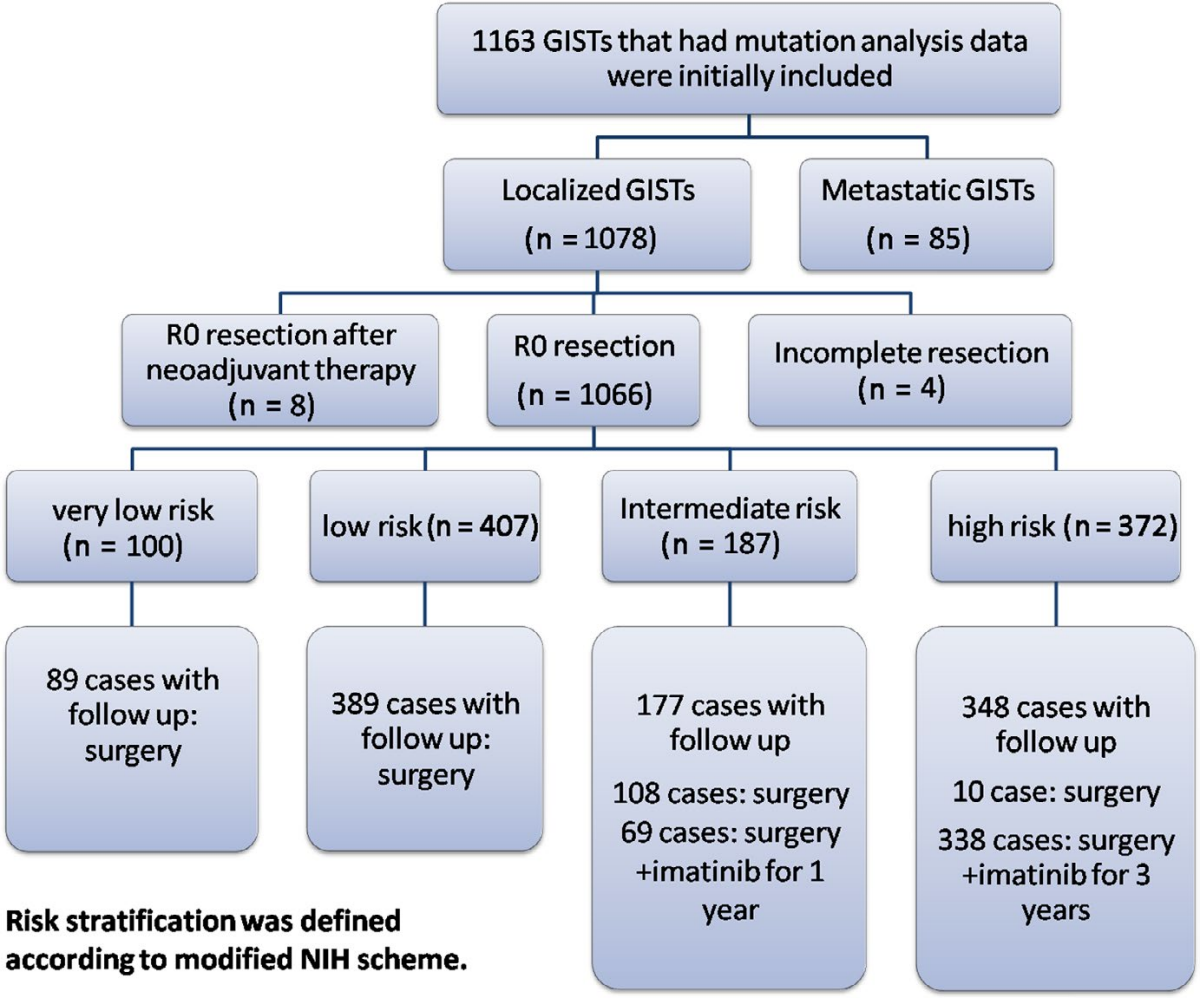
Patients included in the study

Clinicopathological characteristics and mutational status were available for 1163 patients with GIST (629 men and 534 women). The median patient age at the time of GIST detection was 60 years (range, 25‐90 years). The most common tumor site was the stomach (n = 670, 57.6%), followed by the small intestine (n = 391, 33.6%) and rectum (n = 34, 2.9%). Other rare tumor sites and abdominal and pelvic masses of unknown origin were placed in the other groups. There were 1078 cases of localized GISTs and 85 cases of metastatic GISTs at the time of initial diagnosis. Of the 1078 cases of localized GISTs, 4 cases were not completely resected, 1066 cases were treated with complete surgical resection, and 8 cases were treated with complete surgical resection after imatinib neoadjuvant therapy (Figure 1). The median follow‐up time of the surviving patients was 43 months (range: 1‐150 months). The clinicopathological characteristics, including sex, age, tumor site, tumor size, mitosis, and risk assessments, are shown in Table S2.

### 3.2 Genotype analysis

Among the 1,163 cases of GIST, 3.96% (46/1,163) of the cases did not have mutations in the *KIT* or *PDGFRA* gene, that is, the wild type. Eighteen of them are SDH deficient GISTs.

**FIGURE 2:**
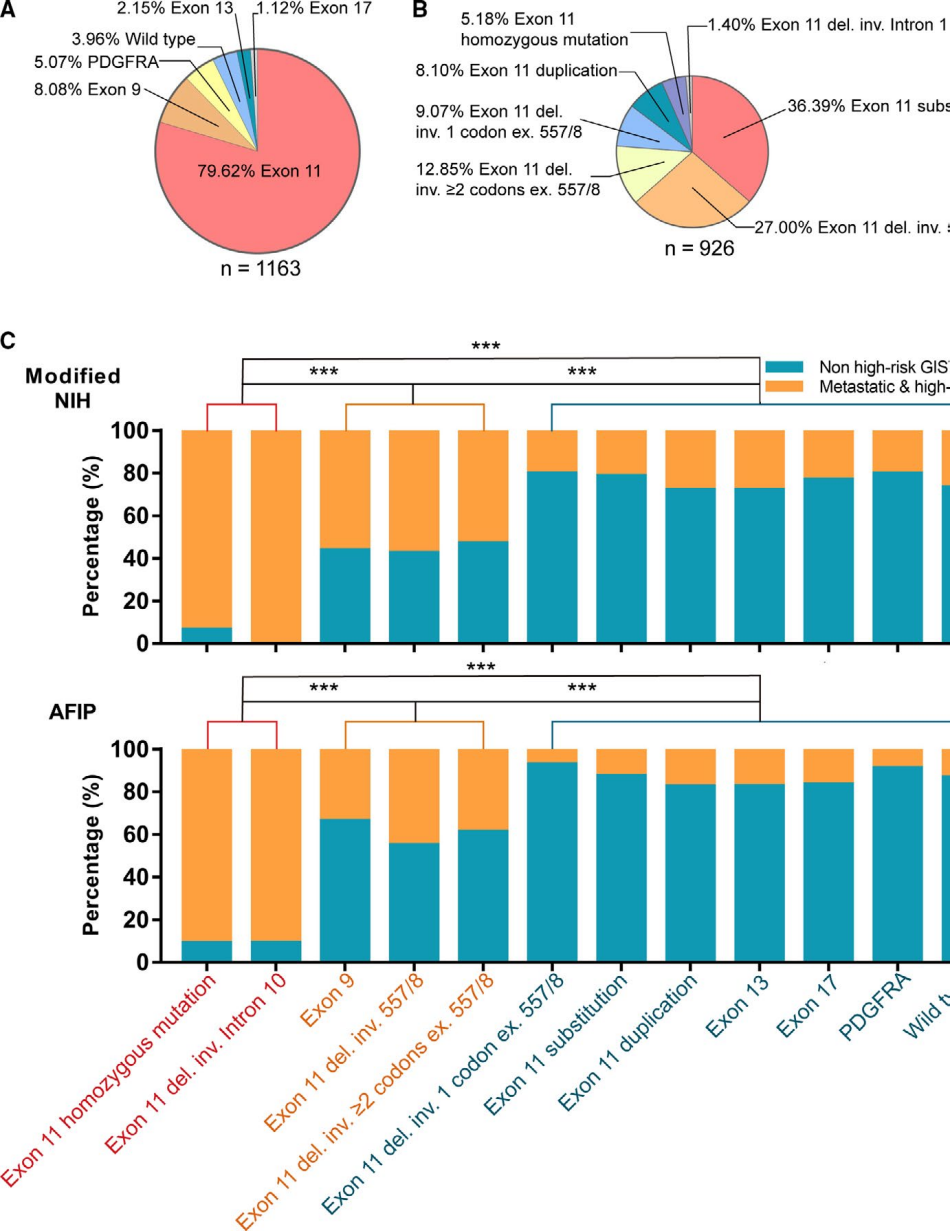
Pie graphs showing the percentage of gastrointestinal stromal tumors (GISTs) classified based on gene mutations. A, Six types of genetic mutations were identified, including wild‐type; *KIT* exon 9, 11, 13, and 17 mutations; and *PDGFRA* mutations. B, The mutation types of *KIT* exon 11 mutation were further divided into seven types. The GISTs were divided into 12 categories according to different mutation types. C, The histogram shows the proportion of high‐risk and metastatic GISTs among the 12 categories of different mutant GISTs. The 12 categories of different mutant GISTs can be divided into three groups according to the proportion of high‐risk and metastatic GISTs. Pairwise comparisons among the three groups showed significant differences. inv.: involving, ex.: excluding; ^***^ *P* < .001

The most frequent mutation in *PDGFRA* was p.D842V (74.6%, 44/59). The most common *KIT* exon 17 mutation was p.N822H (92.3%, 12/13). All 94 cases with *KIT* exon 9 mutation had p.A502_Y503dup. All 25 cases with *KIT* exon 13 mutation resulted in a missense change, p.K642E, and 3 cases had double mutations, showing not only p.K642E, but also p.S639T/p.M651G. All these mutations were heterozygous. All the remaining GISTs carried *KIT* exon 11 mutations. The percentage of cases with these six categories of GISTs with different mutation types is shown in Figure 2A.

**FIGURE 3:**
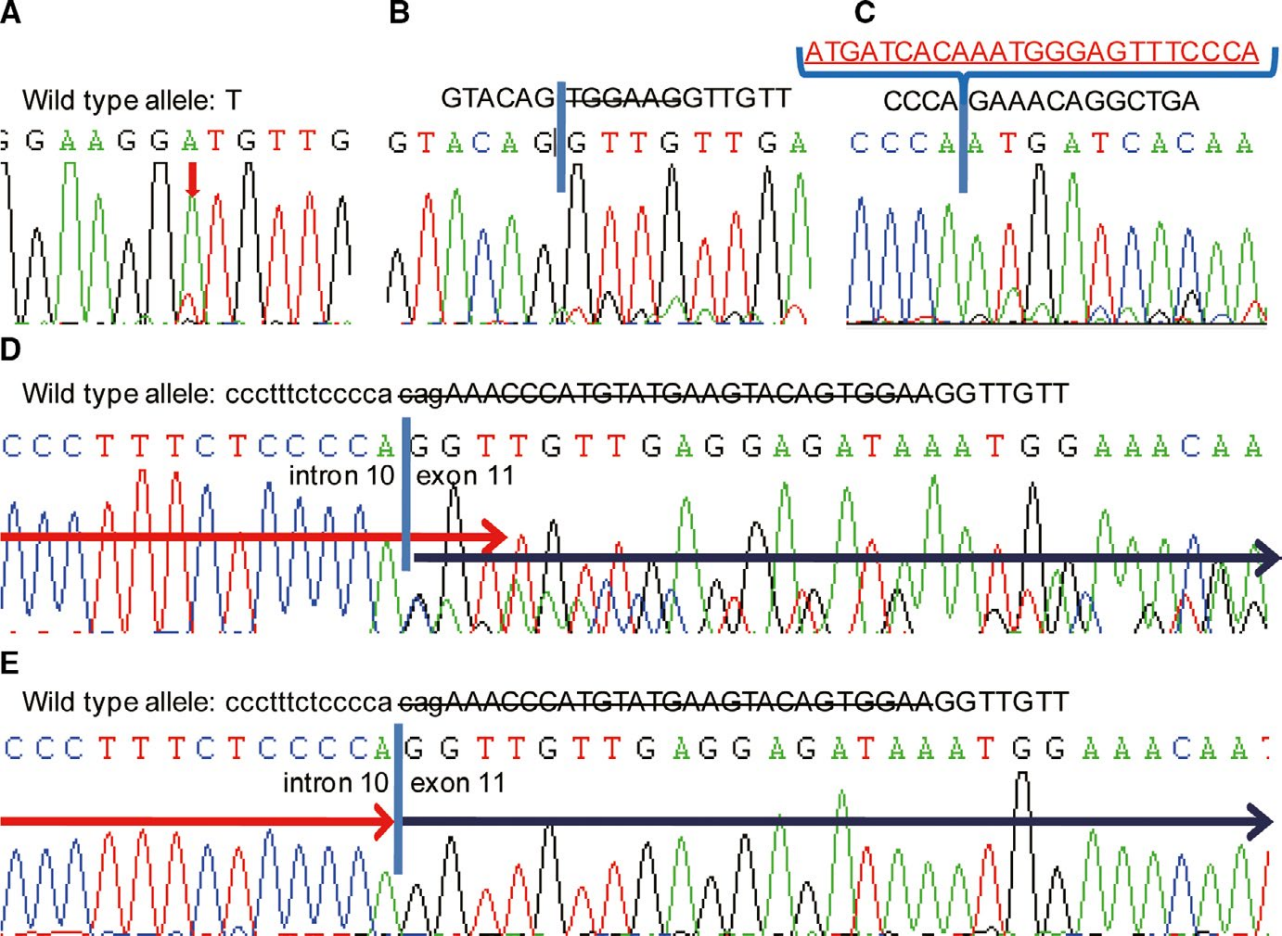
Examples of direct sequencing of *KIT* mutations. A, *KIT* exon 11 homozygous substitution (p.V559D); B, *KIT* exon 11 homozygous deletion (p.W557_K558del); C, *KIT* exon 11 homozygous duplication (p.578_585dup); D, *KIT* heterozygous deletion including intron 10/exon 11 junction; E, *KIT* homozygous deletion, including intron 10/exon 11 junction

*KIT* exon 11 mutations were highly complicated and diverse. Among the 926 cases of GIST with *KIT* exon 11 mutations, 48 cases had homozygous mutations and the remaining cases had heterozygous mutations. *KIT* exon 11 homozygous mutations included substitutions (16 cases; Figure 3A), deletions/indel (31 cases; Figure 3B), and duplications (1 case; Figure 3C). *KIT* exon 11 heterozygous mutations also included substitutions (337 cases, the most frequent types were p.V559X, p.V560X, p.W557X, and p.L576P), deletions/indel (466 cases), and duplications (75 cases). Among the 466 cases of *KIT* exon 11 heterozygous deletion mutations, 13 cases showed deletions involving the intron 10/exon 11 junction (involving codons 550‐558; Figure 3D). In the homozygous mutations, there were two such cases (Figure 3E). Among the remaining heterozygous *KIT* gene deletions, 250 cases had deletions involving the 557 and/or 558 codons. Among the 203 cases with heterozygous *KIT* gene deletion outside codon 557 or 558, 84 cases had deletions involving only one codon (the most frequent types were del559/560, del576, and del579), and 119 cases had deletions involving two or more codons. The percentage of cases with these seven categories of GISTs with different *KIT* exon 11 mutations is shown in Figure 2B.

We classified the gene mutation types into the following 12 categories: wild‐type; *PDGFRA* mutations; *KIT* exon 9 mutations; *KIT* exon 13 mutations; *KIT* exon 17 mutations; *KIT* exon 11 homozygous mutations; *KIT* heterozygous intron 10/exon 11 junction deletions (involving codons 550‐558); other heterozygous *KIT* exon 11 deletions involving codon 557 or 558; other heterozygous *KIT* exon 11 deletions only involving one codon, excluding codon 557 and 558; other heterozygous *KIT* exon 11 deletions involving two or more codons, excluding codons 557 and 558; and *KIT* exon 11 heterozygous substitutions and duplications.

### 3.3 Relationship between gene mutation types and clinicopathological features of GISTs

The relationship between gene mutation types and clinicopathological features of GISTs is presented in Table S2. Age and sex were not significantly different among the 12 gene mutation types (age: *P* = .064; sex: *P* = .288). *KIT* exon 9 duplications were frequently detected in the small intestine (87.2%), whereas *PDGFRA* mutations were mainly associated with the gastric location (89.8%). When we compared the proportions of the 12 different genotypes of GISTs in the high‐risk (according to the modified NIH or AFIP scheme) and metastatic groups, we found that they could be reclassified into the following three groups: Group 1: *KIT* exon 11 homozygous mutations and *KIT* heterozygous intron 10/exon 11 junction deletions (involving codons 550‐558); Group 2: other heterozygous *KIT* mutations, including exon 9 duplications, exon 11 deletions involving codons 557 and/or 558, and deletions involving two or more codons, excluding codons 557 and/or 558; and Group 3: other heterozygous *KIT* exon 11 mutations, including deletions involving only one codon, excluding codon 557 and 558; substitutions and duplications; *KIT* exon 13 and 17 mutations; *PDGFRA* mutations and the wild‐type. Group 1 was the most common among high‐risk and metastatic GISTs, whereas group 3 was observed in low‐risk GISTs. The proportion of group 2 was between that of groups 1 and 3 in high‐risk and metastatic GISTs. Pairwise comparisons among the three groups showed significant differences (Table S3 and Figure 2C). The average primary tumor size in group 1 was 8.5 cm and the mitotic phase count was more than 10/50 HPF in the majority of the cases (32/43, 74.4%). 42 (97.7%) tumors had spindle cell morphology and only one tumor had epithelioid components. Compared with other mutated types of GISTs, the mitotic index of GIST in group 1 was much higher. The tumor size was larger than that of other mutated types of GISTs, except GISTs with KIT exon 11 deletion involving 557/558, which was statistically significant (All of *P* was < .05).

### 3.4 Tumor originating from the rectum and KIT exon 11 deletions involving two or more codons predict worse DFS in patients with low‐risk GIST

In our study, there were 89 cases of very low‐risk GISTs and 389 cases of low‐risk GISTs (with follow‐up data) on using the modified NIH schemes. The information of patients with relapse is shown in Table S4.

**FIGURE 4:**
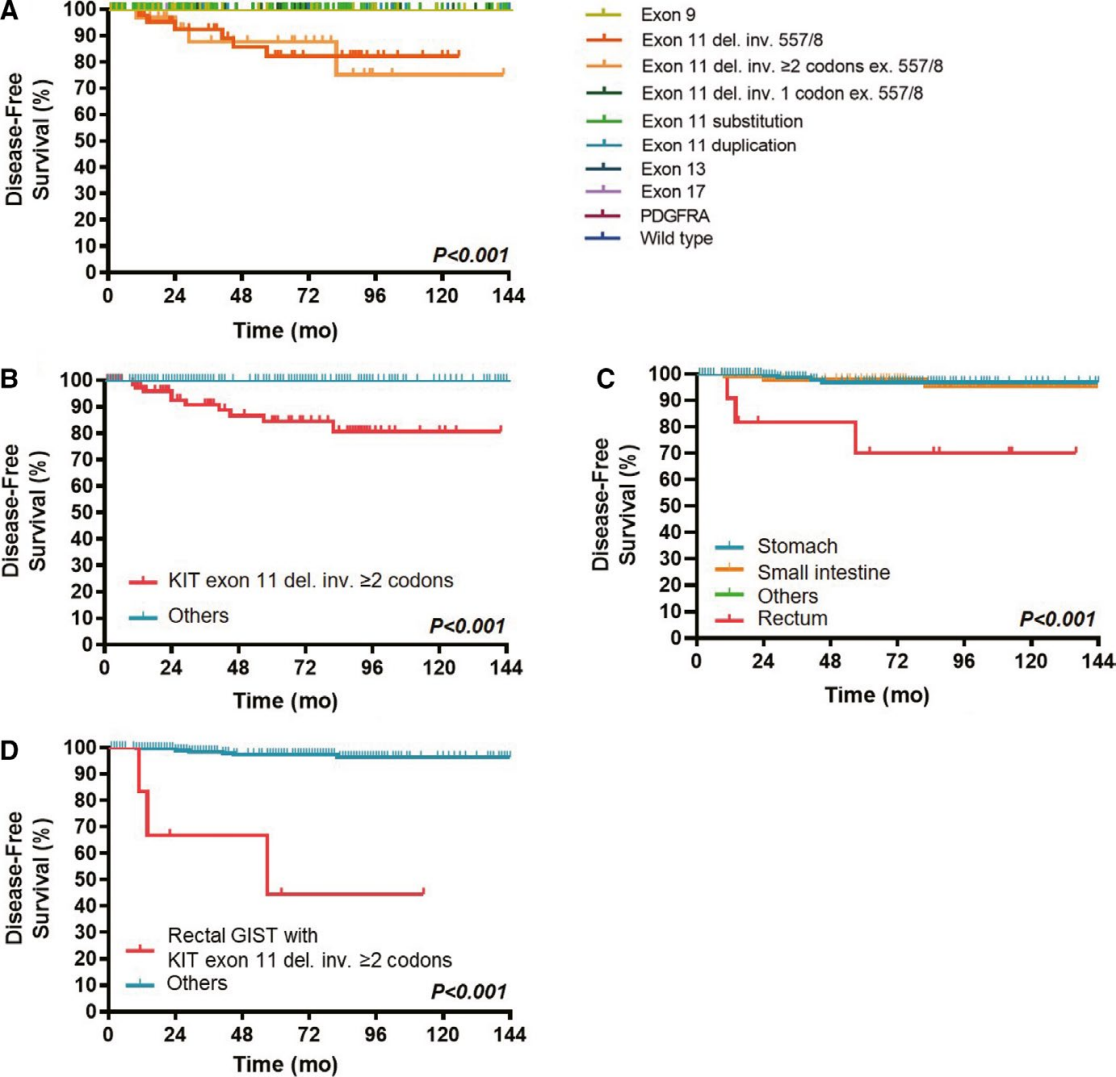
Disease‐free survival (DFS) of patients with low‐risk GISTs according to the type of tumor mutation (a total of 10 categories of different mutant GISTs) (A), mutation status (*KIT* exon 11 deletions involving two or more codons vs others) (B), tumor site (C) and combined factors (rectal GISTs with *KIT* exon 11 deletions involving two or more codons vs others) (D)

The very low‐risk and low‐risk GIST patients underwent complete surgical resection without any adjuvant therapy. In the very low‐risk group, none of the patients experienced recurrence. In the low‐risk group, recurrence occurred in 10 patients. All 10 patients with recurrent GIST harbored *KIT* exon 11 heterozygous deletions involving two or more codons. In six cases, the deletions affected codon 557 or 558; and in the other four cases, the deletions did not affect codon 557 or 558. None of the patients in the very low‐risk and low‐risk groups had *KIT* exon 11 homozygous mutations or deletions involving intron 10. Low‐risk GIST patients with other eight mutation types, including the *KIT* exon 9 mutation, did not recur during follow‐up (Figure 4A). For the initial univariate analysis on DFS, we divided gene mutation types into two groups: *KIT* exon 11 deletions involving two or more codons and others. In the group of low‐risk GISTs with *KIT* exon 11 heterozygous deletions involving two or more codons, the recurrence rate was 11% (10/87). Among these 10 patients with recurrent low‐risk GISTs, the tumors were located in the stomach, small intestine, and rectum in 4, 3, and 3 patients, respectively. The recurrence rates of low‐risk GISTs in the stomach, small intestine, and rectum were 1.6% (4/257), 2.5% (3/119), and 27.3% (3/11), respectively.

On univariate analysis, tumors originating from the rectum or with *KIT* exon 11 deletions involving two or more codons were found to predict worse DFS in low‐risk GIST cases (Figure 4B,C). Moreover, if we combined the two factors mentioned above, the recurrence rate was as high as 50% (3/6) in patients with low‐risk rectal GISTs with *KIT* exon 11 deletions involving two or more codons. The recurrence rate in these patients was also higher than that in the other patients and the difference was statistically significant (Figure 4D). However, on multivariate analysis, neither the tumor site nor the gene mutation type was an independent prognostic factor (Table S5).

### 3.5 Prognostic analysis of patients with intermediate‐risk of GIST

In our study, there were 187 cases of intermediate‐risk of GISTs on using the modified NIH scheme. Among the 177 intermediate‐risk GISTs patients with follow‐up data, 69 patients received imatinib therapy for 1 year in addition to complete surgical resection and 108 patients underwent surgery only. A total of five patients developed recurrence. Two patients received imatinib for one year and the other three did not receive imatinib. The information of patients with relapse is shown in Table S4. One patient had a homozygous mutation (*KIT* p.V555_V560del). Among the other four patients who developed recurrence, one had *PDGFRA* mutation, and the other three had *KIT* exon 11 deletions involving codon 557 or 558 and two or more codons. Similar to the patients in the low‐risk group, none of the patients in the intermediate‐risk group had *KIT* exon 11 deletion, including intron 10, and only 3 patients had homozygous mutations. Because of the small number of cases in this group, statistical analysis was not performed.

### 3.6 Prognostic value of KIT exon 11 homozygous mutations and deletions involving intron 10/exon 11 junction in patients with high‐risk GIST treated with surgery and imatinib therapy

**FIGURE 5:**
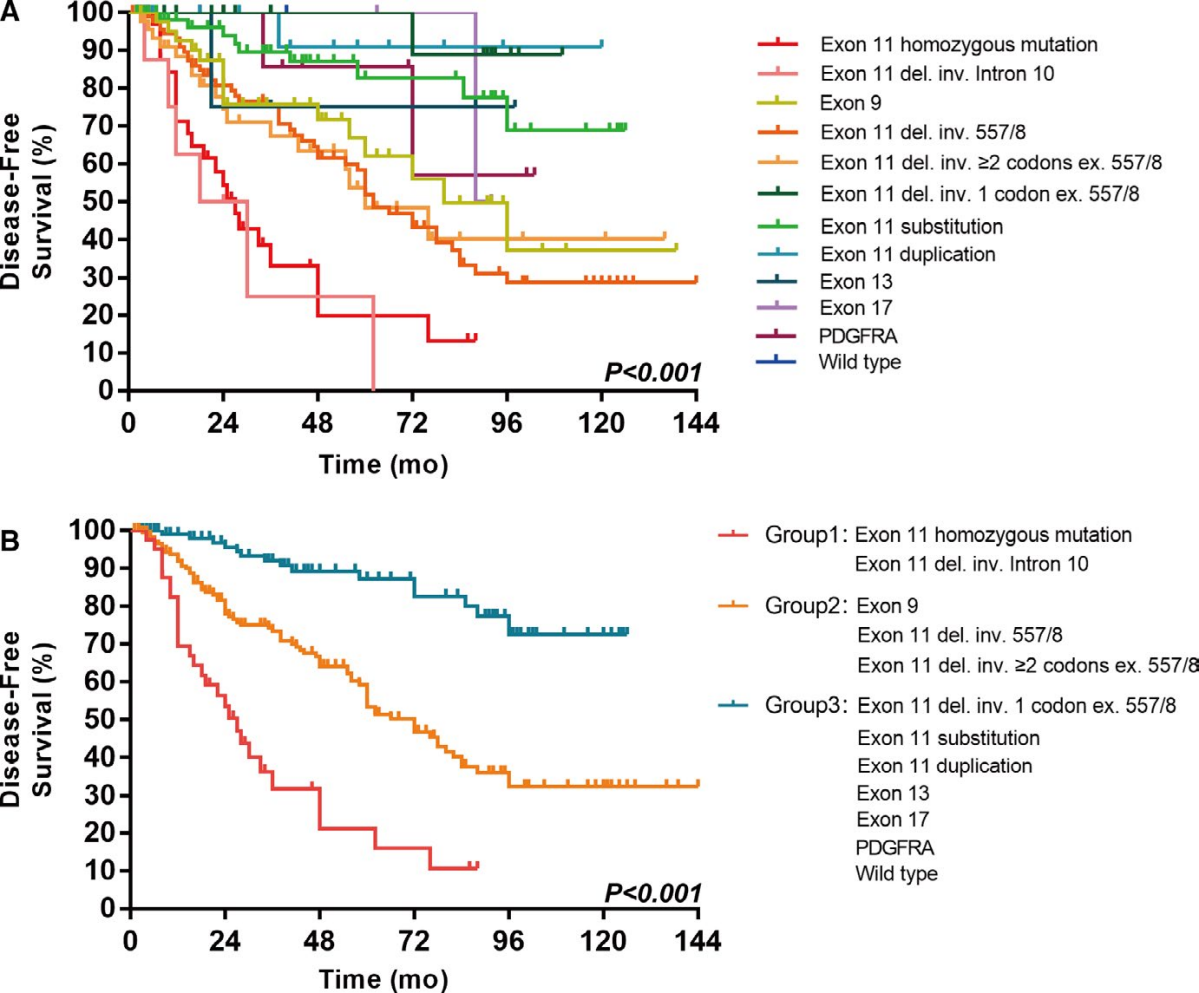
Disease‐free survival (DFS) of 338 patients with high‐risk GISTs according to the type of tumor mutation (a total of 12 categories of different mutant GISTs) (A) and according to mutation status (group 1 vs group 2 vs group 3) (B)

**FIGURE 6:**
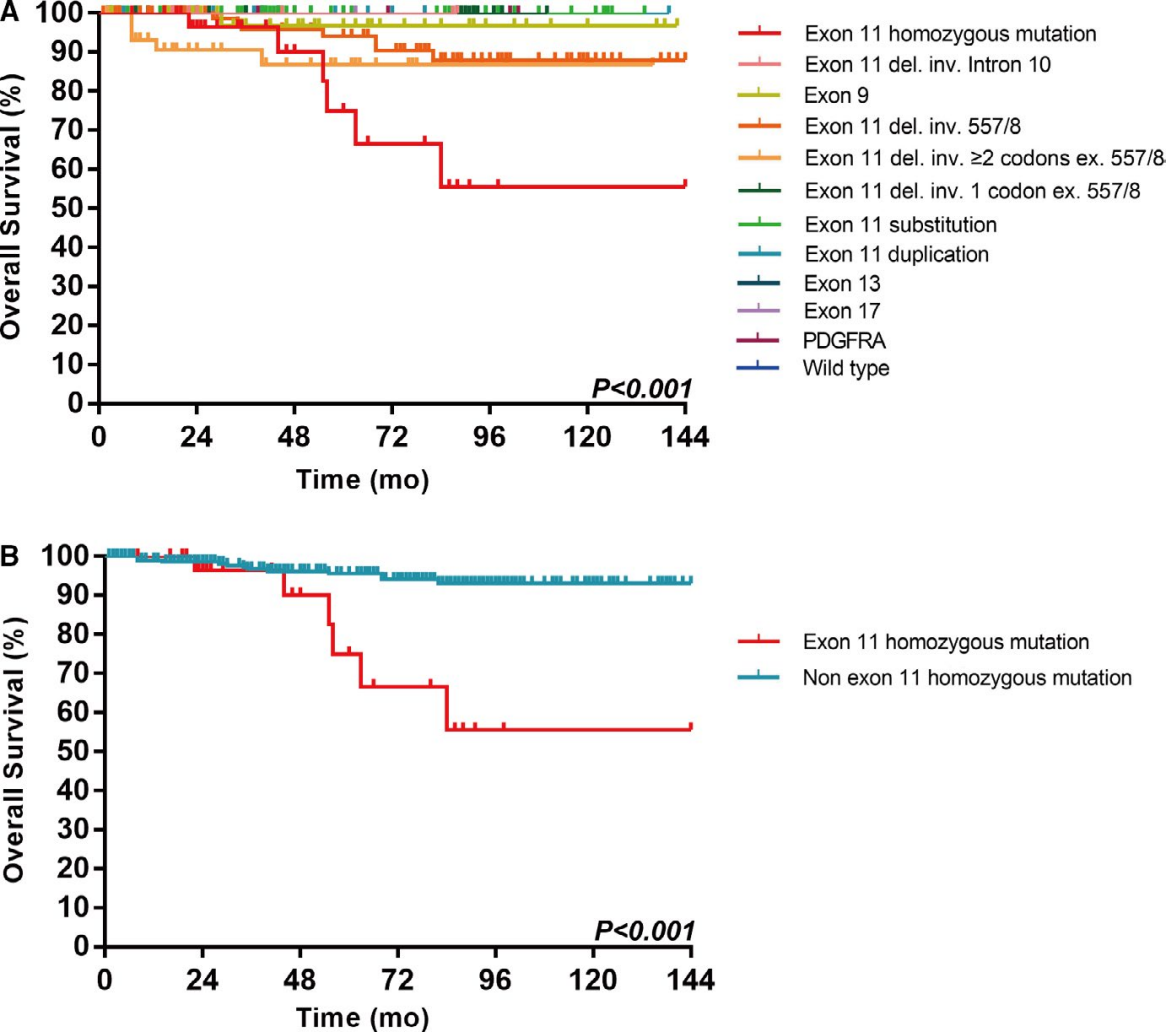
Overall survival (OS) of 338 patients with high‐risk GISTs according to the type of tumor mutation (a total of 12 categories of different mutant GISTs) (A) and according to mutation status (*KIT* exon 11 homozygous mutation vs non‐homozygous mutation) (B)

There were a total of 372 cases of high‐risk GIST according to the modified NIH scheme. Most patients (90.9%, 338/372) received imatinib treatment for 3 years in addition to complete surgical resection, and their follow‐up data were available. During follow‐up, 122 patients had recurrence and 19 died from GIST. Patients with GIST with *KIT* exon 11 homozygous mutations and *KIT* intron 10/exon 11 junction deletions had the highest recurrence rate, which was even worse than that in patients who had GIST with KIT exon 9 duplication or other exon 11 deletions involving codons 557‐558 and exon 11 deletion involving two or more codons (Figure 5A). In addition, patients who had GIST with *KIT* exon 11 homozygous mutations had the most unfavorable OS (Figure 6A).

For the initial univariate analysis on DFS, we also divided gene mutation types into the following three groups. Group 1: *KIT* exon 11 homozygous mutations and *KIT* heterozygous intron 10/exon 11 junction deletions (involving codons 550‐558); Group 2: other heterozygous *KIT* mutations, including exon 9 duplications, exon 11 deletions involving codons 557 and/or 558, and deletions involving two or more codons, excluding codons 557 and/or 558; and Group 3: heterozygous *KIT* exon 11 deletions involving only one codon, excluding codon 557 and 558; substitutions and duplications; *KIT* exon 13 and 17 mutations; *PDGFRA* mutations and the wild‐type. This classification was the same as the previous classification obtained from the chi‐square test evaluating the gene mutation type and risk assessment stratification. There were significant differences in DFS among the three groups (*P* < .001; Figure 5B). Sex, tumor size, mitosis, and mutation status were significant prognostic factors not only on a Cox univariate model but also on a Cox multivariate model for DFS (Table 1).

**TABLE 1:** Univariate and multivariate Cox proportional hazards models to predict factors associated with disease‐free survival (DFS) of patients with high‐risk (modified NIH scheme) gastrointestinal stromal tumors (GISTs) treated with surgery and imatinib therapy

Category	Univariate		Multivariate	
	HR (95% Cl)	*P* value	HR (95% Cl)	*P* value
Age (y)				
≤60	1.0 (reference)		1.0 (reference)	
>60	1.048 (0.734‐1.497)	.797	0.913 (0.621‐1.344)	.646
Sex				
Male	1.0 (reference)		1.0 (reference)	
Female	0.683 (0.472‐0.988)	.043	0.591 (0.404‐0.865)	.007
Tumor site		.634		.103
Stomach	1.0 (reference)		1.0 (reference)	
Small intestine	0.789 (0.531‐1.173)	.241	1.488 (0.958‐2.310)	.077
Rectum	1.069 (0.479‐2.381)	.871	1.209 (0.510‐2.864)	.667
Other	0.952 (0.520‐1.741)	.873	2.158 (1.126‐4.136)	.063
Tumor size		.004		.001
2‐5 cm	1.0 (reference)		1.0 (reference)	
5‐10 cm	0.870 (0.466‐1.626)	.663	2.164 (1.106‐4.233)	.024
>10 cm	1.633 (0.874‐3.052)	.124	3.268 (1.681‐6.353)	<.001
Mitosis		<.001		<.001
≤5/50 HPF	1.0 (reference)		1.0 (reference)	
5‐10/50 HPF	2.380 (1.369‐4.139)	.002	2.788 (1.564‐4.967)	.001
>10/50 HPF	4.955 (2.970‐8.268)	<.001	3.797 (2.118‐6.808)	<.001
Mutation status		<.001		<.001
Group 1^a^	1.0 (reference)		1.0 (reference)	
Group 2^b^	0.364 (0.234‐0.566)	<.001	0.412 (0.256‐0.661)	<.001
Group 3^c^	0.093 (0.049‐0.177)	<.001	0.126 (0.063‐0.254)	<.001

Abbreviations: CI, confidence interval; HPF, high‐power fieldHR, hazard ratio.

^a^ Group 1 included *KIT* exon 11 homozygous mutation and *KIT* heterozygous intron 10/exon 11 junction deletions (affecting codon 550‐558).

^b^ Group 2 included other heterozygous *KIT* mutations including exon 9 duplications, exon 11 deletions involving codons 557 and/or 558, and deletions involving two or more codons, excluding codons 557 and/or 558.

^c^ Group 3 included heterozygous KIT exon 11 deletions involving only one codon, excluding codon 557 and 558; substitutions and duplications; KIT exon 13 and 17 mutations; PDGFRA mutations and the wild‐type.

According to the results of the initial survival analysis on OS, we classified the types of gene mutations of GISTs into *KIT* exon 11 homozygous mutations and non‐*KIT* exon 11 homozygous mutations. Patients with *KIT* exon 11 homozygous mutations had a worse OS (*P* < .001; Figure 6B). Mitosis and the presence of *KIT* exon 11 homozygous mutations were significant prognostic factors on Cox univariate and multivariate analyses. These two factors could independently influence the OS (Table 2).

**TABLE 2:** Univariate and multivariate Cox proportional hazards models to predict factors associated with overall survival (OS) of patients with high‐risk (modified NIH scheme) gastrointestinal stromal tumors (GISTs) treated with surgery and imatinib therapy

Category	Univariate		Multivariate	
	HR (95% Cl)	*P* value	HR (95% Cl)	*P* value
Age (y)				
≤60	1.0 (reference)		1.0 (reference)	
>60	1.354 (0.550‐3.334)	.510	1.047 (0.372‐2.943)	.931
Sex				
Male	1.0 (reference)		1.0 (reference)	
Female	0.845 (0.071‐2.841)	.525	0.821 (0.063‐3.783)	.669
Tumor site		.365		.458
Stomach	1.0 (reference)		1.0 (reference)	
Small intestine	0.412 (0.146‐1.160)	.093	0.738 (0.254‐2.142	.576
Rectum	0.727 (0.092‐5.757)	.763	2.026 (0.218‐18.794)	.534
Other	1.025 (0.277‐3.789)	.971	2.213 (0.515‐9.503)	.285
Tumor size		.153		.421
2‐5 cm	1.0 (reference)		1.0 (reference)	
5‐10 cm	0.385 (0.096‐1.539)	.177	1.019 (0.229‐4.526)	.980
>10 cm	1.000 (0.275‐3.639)	1.000	1.929 (0.484‐7.688)	.352
Mitosis		.003		.024
≤5/50 HPF	1.0 (reference)		1.0 (reference)	
5‐10/50 HPF	3.132 (0.326‐30.123)	.323	3.286 (0.324‐33.347)	.314
>10/50 HPF	15.213 (2.009‐115.175)	.008	11.823 (1.454‐96.113)	.021
Mutation status				
KIT exon 11 homozygous mutation	1.0 (reference)		1.0 (reference)	
Non‐KIT exon 11 homozygous mutation	0.186 (0.070‐0.492)	.001	0.304 (0.097‐0.951)	.041

Abbreviations: CI, confidence interval; HPF, high‐power fieldHR, hazard ratio.

In the high‐risk group, 32 cases of GIST with *KIT* exon 11 homozygous mutations were found. Ten of them had homozygous substitutions. During the follow‐up period, there were 6 cases of recurrence (60%) and 1 case died from GIST (10%). Among the 22 cases of homozygous deletions, 16 cases relapsed (72%) and 6 cases died (27%). There were no homozygous duplications in this group. GIST patients with homozygous deletions seemed to have a worse prognosis than those with homozygous substitutions, but the difference was not statistically significant. (DFS: *P* = .931, OS: *P* = .475).

### 3.7 Prognostic analysis of patients with metastatic GIST at the time of initial diagnosis

In our study, there were 85 cases of metastatic GISTs at the time of initial diagnosis. A total of 19 cases only underwent biopsy without surgery, and 66 cases underwent R_0_/R_1_/R_2_ resection. All patients received targeted therapy, such as imatinib, sunitinib, and regorafenib. Among the 85 cases of metastatic GIST, 13 cases had *KIT* exon 11 homozygous mutations (15.3%, 13/85) and 5 cases had *KIT* intron 10/exon 11 junction deletions (5.9%, 5/85) (Table S2). During the follow‐up period, 5 patients with *KIT* exon 11 homozygous mutations and 2 patients with *KIT* intron 10/exon 11 junction deletions died. Of the 52 GIST patients (with follow‐up data) with other mutations, 8 patients died. These data were not statistically analyzed due to many confounding factors in treatment.

## 4 DISCUSSION

In the current study of 1163 Chinese patients with GIST, most clinicopathological features, such as age, sex, tumor size, location, and mitosis, were similar to those reported in previous studies.^1^, ^4^, ^5^, ^6^, ^7^, ^8^, ^9^, ^10^

In the present study, we first classified the genotypes of GISTs into 12 categories and found that GIST patients with *KIT* homozygous mutations or *KIT* intron 10/exon 11 junction deletions were most commonly found in metastatic and high‐risk GISTs compared to GISTs with other mutation types. In the high‐risk GIST group, the above‐mentioned two groups of GISTs had the highest recurrence rate even after complete surgical resection plus imatinib adjuvant therapy, thereby being independent prognostics factors of DFS. The presence of *KIT* exon 11 homozygous mutations independently influenced the OS. Our results indicated that although the two groups of GISTs were very rare (homozygous *KIT* mutations: 48/1163, 4.1%; *KIT* intron 10/exon 11 junction deletions: 15/1163, 1.3%), they showed a very high degree of invasive biological behavior.

There are only a few reports of homozygous mutated GISTs possibly owing to the very low incidence. The *KIT* homozygous mutation was mentioned in a study with a large sample size of GISTs.^10^ 4% (17/427) of GISTs had *KIT* exon 11 homozygous mutations, including 12 deletions, 4 substitutions, and 1 complex mutation. Most cases (11/17) were high‐risk or disseminated GISTs. Lasota et al reported 36 cases of homozygous mutated GISTs.^12^ In their study, all the homozygous mutations occurred in *KIT* exon 11, including point mutations and deletions/indels. The average tumor size was 12 cm, and the average mitotic activity was 32/50 HPFs. Moreover, 26 of the 29 patients had metastases or died of disease during follow‐up. Similar to our findings, both previous studies showed aggressive biological behavior in GISTs with *KIT* homozygous mutations. Interestingly, Lasota et al also found 2 cases of GISTs with heterozygous mutations in the primary tumor and homozygous mutations in recurrent or metastatic tumors, suggesting an important role of oncogene homozygosity in tumor progression. Furthermore, Chen et al showed that mitotic nondisjunction was the mechanism underlying this shift from heterozygous to homozygous mutations.^14^ Their findings also showed that the percentage of nuclear localization of pY568KIT (Tyr568 of KIT is the initial and primary autophosphorylation site of KIT) and topoisomerase II proliferative index increased fourfold in the homozygous GISTs compared to the heterozygous counterpart within the same patients. These may result in an aggressive phenotype during evolution from heterozygous to homozygous KIT‐activating mutation.^14^ Chen et al thought the following model can be used to explain this phenomenon. Upon ligand binding, KIT first forms a homodimer, followed by autophosphorylation of several tyrosine residues of KIT. Homodimers containing mutated KIT in both alleles likely contribute to the majority of the constitutive proliferative signal in GISTs. When GIST exhibits a heterozygous KIT mutation, 50% of the homodimers are paired with one mutated KIT and one wild‐type KIT, and only “25%” of homodimers are paired with both mutated KIT. In contrast, when the GIST evolves to a homozygous KIT mutation, “100%” of homodimers are paired with the mutated KIT, and they can potentially transmit a fourfold increase in KIT signaling compared to the same patient's heterozygous counterpart.

In addition, we compared the prognosis of GIST patients with *KIT* exon 11 homozygous deletions and homozygous substitutions for the first time. GIST patients with homozygous deletions seemed to have a worse prognosis than those with homozygous substitutions, but the difference was not statistically significant. More cases are needed to determine whether there was a difference in prognosis between the two homozygous mutant GISTs.

*KIT* deletions involving the intron 10/exon 11 junction have been reported in three previous studies.^10^, ^11^ In a clinical study of 427 tumors in Poland, 6 tumors (1.4%) were detected with this particular mutation.^10^ Among 722 cases of GIST in Americans, 19 GISTs (2.6%) carried the splice site deletion.^11^ Similar to the results of the current study, the results of both studies showed that most GISTs with the rare *KIT* mutation were rated as high‐risk (Poland: 5/6; America: 16/19). However, this particular mutation appeared to occur at a high rate in the Arab population.^13^ Among 52 GISTs, 19 cases (36.5%) were identified with splice site deletion of *KIT*. One possibility is that the incidence of this particular mutation might vary among populations. Another possibility is that the number of cases in the study performed in the Arab population was too low to obtain precise incidence data. In our study, *KIT* deletions involving the intron 10/exon 11 junction result in loss of codon 550‐558 (Figure S1). It should be noted that the deletion codon 550‐558 can also result from an in‐frame deletion that begins precisely at the first coding nucleotide of exon 11 (codon 550). We found three cases of such deletions in our study. Two cases were assessed as high risk and one case was assessed as low risk. One patient had relapsed during the follow‐up. At the protein level, the final mutant isoform is the same as in tumors that have the deleted part of intron 10 and use the alternative splice acceptor. Whether or not the prognosis of GIST with these two gene mutation types is equally poor needs further confirmation.

Although previous studies and our study confirmed that GISTs with *KIT* homozygous mutations and exon 11/intron 10 junction deletions were highly invasive, only two studies have reported the effect of imatinib on these particular mutations. In the study by Lasota et al, 8 patients with advanced GIST with homozygous mutations were treated with imatinib only. Initially, 6 cases and 1 case showed a partial response (PR) and stable disease (SD), respectively.^12^ However, 4 patients developed progressive disease immediately after brief periods of remission (5 months in 3 patients and 2 months in 1 patient). A secondary mutation (V654A) was identified in 1 patient who was resistant to imatinib. Corless et al have previously reported the response of GIST patients with *KIT* exon 11/intron 10 junction deletion to imatinib.^11^ In their study, among 6 patients treated with imatinib for advanced disease, 5 achieved PR and 1 showed SD. In the current study, 1 GIST patient with *KIT* exon 11/intron 10 junction deletion underwent surgery after 6 months of neoadjuvant imatinib therapy. Postoperative pathological examination revealed that the tumor cell density decreased significantly along with extensive interstitial collagenzation, inflammatory cell infiltration, and histiocytic reaction (data not shown). Moreover, in the present study, the DFS of GIST patients with the two particular mutation types (KIT exon 11 homozygous mutations and intron 10/exon 11 junction deletions) was very short. The DFS of most of the patients with recurrence was < 36 months, that is, recurrence in most cases occurred during imatinib treatment. These results suggest that imatinib is effective against both types of mutant GISTs, although these GISTs rapidly became resistant and eventually showed recurrence.

In the classification of the 12 gene mutation types, we found that patients with *KIT* exon 11 deletion involving only one codon had a better prognosis than those with other *KIT* exon 11 deletions. GISTs with *KIT* exon 11 deletion involving only one codon were rarely evaluated as high‐risk or metastatic. Only one previous study has identified this mutant GIST and analyzed it separately. In the study by Joensuu et al, GISTs with deletions involving a single codon had smaller tumors, lower mitotic counts, and more favorable DFS compared to those with larger deletions.^16^ In the current study, high‐risk and metastatic GISTs were more common in group 2 and had worse DFS compared with group 3, similar to the results reported in previous studies.^6^, ^7^, ^8^, ^9^, ^10^, ^17^

The present study is the first to show the effect of different gene mutation types on prognosis in different risk groups. In the low‐risk GIST group, patients with rectal tumors or *KIT* exon 11 deletions involving two or more codons had relatively high recurrence rates. Although both the tumor site and the gene type were associated with prognosis on univariate analysis, neither of the two factors were independent prognostic factors on multivariate analysis, probably owing to the insufficient sample size. Therefore, it remains to be studied whether we underestimated the risk in these GIST patients.

## 5 CONCLUSIONS

In summary, low‐incidence mutation subtypes such as *KIT* exon 11 homozygous mutations or *KIT* intron 10/exon 11 junction deletions in GISTs should be carefully considered to explore new treatment strategies in the clinic, as tumors with these mutations have a high recurrence rate and a very poor prognosis after surgery followed by imatinib adjuvant treatment.

## CONFLICT OF INTEREST

The authors declare that they have no competing interests.

## AUTHOR CONTRIBUTIONS

Yan‐Ying Shen and Xin‐Li Ma: Conceptualization, validation, investigation, data curation, writing of original draft, writing and editing, visualization, and project administration; Ming Wang, Chun Zhuang, Bo Ni, and Lin Tu: Software, validation, formal analysis, investigation, resources, and data curation; Qiang Liu: Pathological diagnosis; and Wen‐Yi Zhao and Hui Cao: Writing and review, editing, supervision, and funding.

## Supporting information

Fig S1

Table S1

Table S2

Table S3

Table S4

Table S5

## Data Availability

Additional supporting information can be found online in the Supporting Information section.
