# Supplementary material for: Exon 11 homozygous mutations and intron 10/exon 11 junction deletions in the KIT gene are associated with poor prognosis of patients with gastrointestinal stromal tumors
Source: Cancer Med. 2020 Jul 22;9(18):6485–96. doi: 10.1002/cam4.3212 (PMC7520349; doi:10.1002/cam4.3212)
Supplement: Supplementary file 1 — Fig S1 [file CAM4-9-6485-s001.docx]

Supplementary Fig. 1


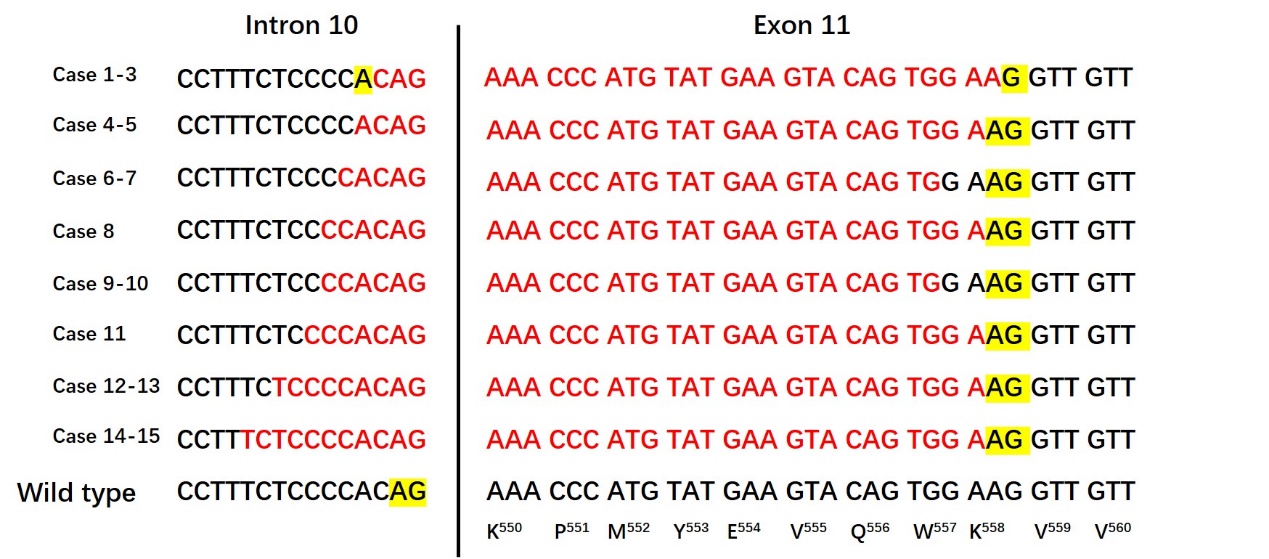


There were 15 cases showed *KIT* deletions involving the intron 10/exon 11 junction in our study (13 cases were heterozygous, and 2 cases were homozygous). As illustrated in the figure, these deletions marked in red included from 1 to 11 bp of the 3’ end of intron 10 and resulted in loss of the splice acceptor site for exon 11. In each case, a new potential splice acceptor AG was created, based on either juxtaposition of an intron 10 adenosine with the terminal guanine of this codon (cases 1 to 3) or the last two nucleotides of codon K558 (cases 4 to 15). Predicted splice acceptor sites were marked in yellow. Therefore, such deletion resulted in the deletion mutation of codon 550–558.
