# Supplementary material for: Exon 11 homozygous mutations and intron 10/exon 11 junction deletions in the KIT gene are associated with poor prognosis of patients with gastrointestinal stromal tumors
Source: Cancer Med. 2020 Jul 22;9(18):6485–96. doi: 10.1002/cam4.3212 (PMC7520349; doi:10.1002/cam4.3212)
Supplement: Supplementary file 2 — Table S1 [file CAM4-9-6485-s002.docx]

Supplementary Table S1-1. Primer sequence

| gene | exon | Primer sequence |
| --- | --- | --- |
| *KIT* | exon 9 | forward: GCCACATCCCAAGTGTTTTATG |
|  |  | reverse: GAGCCTAAACATCCCCTTAAATTG |
| *KIT* | exon 11 | forward: CCAGAGTGCTCTAATGACTG |
|  |  | reverse: TTATGTGTACCCAAAAAGGTGACA |
| *KIT* | exon 13 | forward: GCTTGACATCAGTTTGCCAG |
|  |  | reverse: GACAGACAATAAAAGGCAGCTTG |
| *KIT* | exon 17 | forward: TGGTTTTCTTTTCTCCTCCAACCT |
|  |  | reverse: TGCAGGACTGTCAAGCAGAG |
| *PDGFRA* | exon 12 | forward: CTCTGGTGCACTGGGACTTT |
|  |  | reverse: GCAAGGGAAAAGGGAGTCTT |
| *PDGFRA* | exon 18 | forward: TCAGCCAGTCTTGCAGGGGTGA |
|  |  | reverse: AGGAGGATGAGCCTGACCAGTG |

Supplementary Table S1-2. PCR reaction system

| 2×PCR Master Mix | 12.5μl |
| --- | --- |
| Primer forward  reverse | 1μl  1μl |
| DNA | 1.5~2.5μl |
| H_2_O | 8~9μl |
|  | total 25μl |

Supplementary Table S1-3. PCR reaction conditions

|  | Temperature | time |
| --- | --- | --- |
| Step 1 | 95℃ | 5min |
| Step 2 | 95℃ | 30s |
| Step 3 | 55℃ (*KIT* exon 17) | 30s |
|  | 60℃ (other exons) | 30s |
| Step 4 | 72℃ | 30s |
| Step 5 Repeat Step2~Step4 35 cycles | | |
| Step 6 | 72℃ | 7min |
| Step 7 | 4℃ | forever |
