# Supplementary material for: Exon 11 homozygous mutations and intron 10/exon 11 junction deletions in the KIT gene are associated with poor prognosis of patients with gastrointestinal stromal tumors
Source: Cancer Med. 2020 Jul 22;9(18):6485–96. doi: 10.1002/cam4.3212 (PMC7520349; doi:10.1002/cam4.3212)
Supplement: Supplementary file 3 — Table S2 [file CAM4-9-6485-s003.docx]

Supplementary Table S2. Association between gene mutation types and clinicopathological features of gastrointestinal stromal tumors (GISTs)

|  | Wild type | *PDGFRA*  mutation | *KIT* exon 9  mutation | *KIT* exon 13  mutation | *KIT* exon 17 mutation | *KIT* exon 11 mutation | | | | | | | Total |
| --- | --- | --- | --- | --- | --- | --- | --- | --- | --- | --- | --- | --- | --- |
|  |  |  |  |  |  | homozygous mutation | del.^†^ inv. intron 10 | del.^‡^  inv.557/8 | del.^§^ inv. 1 codon ex.557/8 | del.^¶^ inv.≥2 codons ex.557/8 | substitution | duplication |  |
| Age(years) |  |  |  |  |  |  |  |  |  |  |  |  |  |
| ≤60 | 24 | 28 | 52 | 12 | 4 | 20 | 11 | 131 | 30 | 59 | 162 | 40 | 573 |
| >60 | 22 | 31 | 42 | 13 | 9 | 28 | 2 | 119 | 54 | 60 | 175 | 35 | 590 |
| Sex |  |  |  |  |  |  |  |  |  |  |  |  |  |
| Male | 20 | 39 | 58 | 16 | 7 | 29 | 6 | 144 | 46 | 64 | 171 | 37 | 629 |
| Female | 26 | 20 | 36 | 9 | 6 | 19 | 7 | 106 | 38 | 55 | 166 | 38 | 534 |
| Tumor site |  |  |  |  |  |  |  |  |  |  |  |  |  |
| Stomach | 26 | 53 | 7 | 5 | 7 | 22 | 8 | 135 | 64 | 49 | 236 | 58 | 670 |
| Small intestine | 20 | 2 | 82 | 16 | 4 | 18 | 2 | 73 | 17 | 64 | 79 | 14 | 391 |
| Rectum | 0 | 3 | 1 | 1 | 1 | 3 | 1 | 11 | 0 | 4 | 9 | 0 | 34 |
| Other | 0 | 1 | 4 | 3 | 1 | 5 | 2 | 31 | 3 | 2 | 14 | 2 | 68 |
| Tumor size |  |  |  |  |  |  |  |  |  |  |  |  |  |
| ≤2cm | 9 | 5 | 4 | 6 | 3 | 1 | 0 | 20 | 9 | 7 | 47 | 3 | 114 |
| 2-5cm | 15 | 22 | 43 | 11 | 5 | 7 | 2 | 77 | 42 | 41 | 156 | 35 | 456 |
| 5-10cm | 14 | 26 | 28 | 5 | 3 | 16 | 4 | 72 | 31 | 37 | 104 | 23 | 363 |
| >10cm | 4 | 6 | 10 | 1 | 2 | 11 | 2 | 44 | 1 | 20 | 20 | 12 | 133 |
| Mitosis |  |  |  |  |  |  |  |  |  |  |  |  |  |
| ≤5/50HPF | 38 | 53 | 64 | 20 | 12 | 4 | 0 | 121 | 78 | 74 | 283 | 60 | 807 |
| 5-10/50HPF | 3 | 5 | 15 | 2 | 1 | 6 | 1 | 34 | 4 | 19 | 36 | 11 | 137 |
| >10/50HPF | 1 | 1 | 6 | 1 | 0 | 25 | 7 | 58 | 1 | 12 | 8 | 2 | 122 |

HPF, high-power field

Continued on the following page

Supplementary Table S1. Association between gene mutation types and clinicopathological features of gastrointestinal stromal tumors (GISTs)

|  | Wild type | *PDGFRA*  mutation | *KIT* exon 9  mutation | *KIT* exon 13  mutation | *KIT* exon 17 mutation | *KIT* exon 11 mutation | | | | | | | Total |
| --- | --- | --- | --- | --- | --- | --- | --- | --- | --- | --- | --- | --- | --- |
|  |  |  |  |  |  | homozygous mutation | del.^†^ including intron 10 | del.^‡^ involving 557&/558 | del.^§^ involving 1 codon | del.^¶^ involving≥2 codons | substitution | duplication |  |
| Localized GIST | 43 | 59 | 85 | 23 | 13 | 35 | 8 | 220 | 83 | 107 | 329 | 73 | 1078 |
| Metastatic GIST | 3 | 0 | 9 | 2 | 0 | 13 | 5 | 30 | 1 | 12 | 8 | 2 | 85 |
| NIH | | | | | | | | | | | | | |
| Very low risk | 8 | 2 | 4 | 4 | 3 | 0 | 0 | 17 | 8 | 6 | 45 | 3 | 100 |
| Low risk | 15 | 25 | 37 | 13 | 5 | 0 | 0 | 54 | 43 | 38 | 143 | 34 | 407 |
| Intermediate risk | 10 | 20 | 0 | 1 | 2 | 3 | 0 | 32 | 16 | 11 | 75 | 17 | 187 |
| High risk | 9 | 12 | 44 | 5 | 3 | 32 | 8 | 110 | 16 | 50 | 64 | 19 | 372 |
| AFIP scheme | | | | | | | | | | | | | |
| Benign | 8 | 2 | 4 | 5 | 3 | 0 | 0 | 17 | 8 | 6 | 42 | 3 | 98 |
| Very low risk | 10 | 24 | 5 | 1 | 2 | 0 | 0 | 29 | 35 | 18 | 104 | 24 | 252 |
| Low risk | 14 | 21 | 32 | 11 | 5 | 1 | 0 | 47 | 23 | 31 | 107 | 27 | 319 |
| Intermediate risk | 6 | 3 | 18 | 2 | 0 | 3 | 1 | 30 | 10 | 13 | 29 | 7 | 122 |
| High risk | 4 | 5 | 22 | 3 | 2 | 29 | 6 | 80 | 5 | 35 | 35 | 12 | 238 |

NIH, Risk stratification was performed according to the modified NIH scheme. AFIP, Risk stratification was performed according to the AFIP scheme.

†: *KIT* exon 11 heterozygous deletion involving the intron 10/exon 11 junction (affecting codon 550-558)

‡: *KIT* exon 11 heterozygous deletions involving the 557 and/or 558 codons

§: *KIT* exon 11 heterozygous deletions involving one codon, excluding codons 557 and 558

¶: *KIT*exon 11 heterozygous deletions involving two or more codons, excluding codons 557 and 558
