# Supplementary material for: Exon 11 homozygous mutations and intron 10/exon 11 junction deletions in the KIT gene are associated with poor prognosis of patients with gastrointestinal stromal tumors
Source: Cancer Med. 2020 Jul 22;9(18):6485–96. doi: 10.1002/cam4.3212 (PMC7520349; doi:10.1002/cam4.3212)
Supplement: Supplementary file 4 — Table S3 [file CAM4-9-6485-s004.docx]

Supplementary Table S3.The relationship between gene mutation types and risk assessments of gastrointestinal stromal tumors (GISTs)

|  | *KIT* exon 11 mutation | | *KIT* exon 9  mutation | *KIT* exon 11 mutation | | | | | *KIT* exon 13  mutation | *KIT* exon 17 mutation | *PDGFRA*  mutation | Wild type | Total |
| --- | --- | --- | --- | --- | --- | --- | --- | --- | --- | --- | --- | --- | --- |
|  | homozygous mutation | del.^†^ inv. intron 10 |  | del.^‡^ inv. 557/8 | del.^¶^ inv. ≥2 codons ex.557/8 | del.^§^ inv. 1 codon ex.557/8 | substitution | duplication |  |  |  |  |  |
| NIH |  |  |  |  |  |  |  |  |  |  |  |  |  |
| Non high-risk GISTs | 3  (6.3%) | 0  (0%) | 41  (43.6%) | 103  (42.4%) | 55  (47.0 %) | 67  (79.8%) | 263  (78.5%) | 54  (72%) | 18  (72%) | 10  (76.9%) | 47  (79.7%) | 33  (73.3%) | 694 |
| Metastatic &  high-risk GISTs | 45  (93.7%) | 13  (100%) | 53  (56.4%) | 140  (57.6%) | 62  (53.0%) | 17  (20.2%) | 72  (21.5%) | 21  (28%) | 7  (28%) | 3  (23.1%) | 12  (20.3%) | 12  (26.7%) | 457 |
| AFIP |  |  |  |  |  |  |  |  |  |  |  |  |  |
| Non high-risk GISTs | 4  (9.0%) | 1  (9.1%) | 59  (66.3%) | 124  (54.9%) | 69  (61.1%) | 76  (92.7%) | 282  (87.3%) | 61  (82.4%) | 19  (82.6%) | 10  (83.3%) | 50  (90.9%) | 39  (86.7%) | 794 |
| Metastatic &  high-risk GISTs | 41  (91.0%) | 10  (90.9%) | 30  (33.7%) | 102  (45.1%) | 44  (38.9%) | 6  (7.3%) | 41  (12.7%) | 13  (17.6%) | 4  (17.4%) | 2  (16.7%) | 5  (9.1%) | 6  (13.3%) | 304 |

NIH, Risk stratification was performed according to the modified NIH scheme. AFIP, Risk stratification was performed according to the AFIP scheme.

†: *KIT* exon 11 heterozygous deletion involving the intron 10/exon 11 junction (affecting codon 550-558)

‡: *KIT* exon 11 heterozygous deletions involving the 557 and/or 558 codons

§: *KIT* exon 11 heterozygous deletions involving one codon, excluding codons 557 and 558

¶: *KIT* exon 11 heterozygous deletions involving two or more codons, excluding codons 557 and 558
