# Supplementary material for: Exon 11 homozygous mutations and intron 10/exon 11 junction deletions in the KIT gene are associated with poor prognosis of patients with gastrointestinal stromal tumors
Source: Cancer Med. 2020 Jul 22;9(18):6485–96. doi: 10.1002/cam4.3212 (PMC7520349; doi:10.1002/cam4.3212)
Supplement: Supplementary file 5 — Table S4 [file CAM4-9-6485-s005.docx]

Supplementary Table S4. The detailed information of relapsed patients in low-risk and intermediate-risk gastrointestinal stromal tumors (GISTs)

|  | Tumor site | Tumor size | Mitosis | NIH | AFIP | Imatinib therapy | Time to recurrence | Mutation status | | Homozygous or heterozygous |
| --- | --- | --- | --- | --- | --- | --- | --- | --- | --- | --- |
| Case1 | Stomach | 5cm | <5/50HPF | Low risk | Very low risk | No | 41 months | *KIT*: p.M552_W557indelR | heterozygous | |
| Case2 | Stomach | 4cm | <5/50HPF | Low risk | Very low risk | No | 24 months | *KIT*: p.W557_E561del | heterozygous | |
| Case3 | Stomach | 4.5cm | <5/50HPF | Low risk | Very low risk | No | 45 months | *KIT*: p.W557_K558del | heterozygous | |
| Case4 | Stomach | 4cm | <5/50HPF | Low risk | Very low risk | No | 29 months | *KIT*: p.V559_G565del | heterozygous | |
| Case5 | Rectum | 4cm | <5/50HPF | Low risk | Low risk | No | 11 months | *KIT*: p.W557_V560indelC | heterozygous | |
| Case6 | Rectum | 5cm | <5/50HPF | Low risk | Low risk | No | 14 months | *KIT*: p.Q556_V559del | heterozygous | |
| Case7 | Rectum | 4.5cm | <5/50HPF | Low risk | Low risk | No | 57 months | *KIT*: p.W557_K558del | heterozygous | |
| Case8 | Small intestine | 3cm | <5/50HPF | Low risk | Low risk | No | 82 months | *KIT*: p.V568_L576del | heterozygous | |
| Case9 | Small intestine | 4cm | <5/50HPF | Low risk | Low risk | No | 24 months | *KIT*: p.V559_P573indelA | heterozygous | |
| Case10 | Small intestine | 5cm | <5/50HPF | Low risk | Low risk | No | 10 months | *KIT*: p.V559_V560del | heterozygous | |
| Case11 | Stomach | 8cm | <5/50HPF | Intermediate risk | Low risk | Yes | 19 months | *KIT*: p.W557_K558del | heterozygous | |
| Case12 | Stomach | 9cm | <5/50HPF | Intermediate risk | Low risk | No | 24 months | *PDGFRA*: p.D842V | heterozygous | |
| Case13 | Stomach | 5cm | 6~10/50HPF | Intermediate risk | Intermediate risk | No | 34 months | *KIT*: p.W557_V560indelC | heterozygous | |
| Case14 | Stomach | 4cm | 6~10/50HPF | Intermediate risk | Intermediate risk | No | 66 months | *KIT*: p.E554_K558del | heterozygous | |
| Case15 | Rectum | 2cm | 6~10/50HPF | Intermediate risk | High risk | Yes | 15 months | *KIT*: p.E554_V560del | homozygous | |

HPF, high-power field

NIH, Risk stratification was performed according to the modified NIH scheme.

AFIP, Risk stratification was performed according to the AFIP scheme.
