# Supplementary material for: Exon 11 homozygous mutations and intron 10/exon 11 junction deletions in the KIT gene are associated with poor prognosis of patients with gastrointestinal stromal tumors
Source: Cancer Med. 2020 Jul 22;9(18):6485–96. doi: 10.1002/cam4.3212 (PMC7520349; doi:10.1002/cam4.3212)
Supplement: Supplementary file 6 — Table S5 [file CAM4-9-6485-s006.docx]

Supplementary Table S5. Univariate and multivariate Cox proportional hazards model to predict factors associated with disease-free survival (DFS) of patients with low-risk (modified NIH scheme) gastrointestinal stromal tumors (GISTs) treated with surgery only.

|  | Univariate | | Multivariate | |
| --- | --- | --- | --- | --- |
| Category | HR (95% Cl) | P value | HR (95% Cl) | P value |
| Age(years) |  |  |  |  |
| ≤60 | 1.0 (reference) |  | 1.0 (reference) |  |
| >60 | 0.454 (0.117–1.763) | 0.254 | 1.037 (0.203–5.306) | 0.966 |
| Sex |  |  |  |  |
| Male | 1.0 (reference) |  | 1.0 (reference) |  |
| Female | 0.861 (0.243–3.053) | 0.817 | 1.289 (0.271–6.132) | 0.750 |
| Tumor site |  | 0.005 |  | 0.157 |
| Stomach | 1.0 (reference) |  | 1.0 (reference) |  |
| Small intestine | 1.357 (0.303–6.076) | 0.690 | 1.495 (0.239–9.332) | 0.667 |
| Rectum | 13.264 (2.947–59.693) | 0.001 | 8.191 (1.032–65.021) | 0.053 |
| Others | 0.000 (0.000– -) | 0.991 | 0.000 (0.000–4.316E90) | 1.000 |
| Mutation status |  |  |  |  |
| *KIT* exon 11 del. inv.≥2 codons | 1.0 (reference) |  | 1.0 (reference) | 0.000 |
| Others | 0.002 (0.000–7.655) | 0.136 | 0.000 (0.000–2.687E90) | 0.910 |

HR, hazard ratio; CI, confidence interval

Comment: In the low-risk group, all the tumors were between 2 and 5.0 in size, and the mitotic count was less than 5/50high-power field. Univariate analyses of tumor size and mitotic count were not performed.
